# Supplementary material for: Brain endothelial Gαq/11 signalling in cerebrovascular function and cognition of aged mice
Source: eBioMedicine. 2026 May 18;128:106283. doi: 10.1016/j.ebiom.2026.106283 (PMC13202589; doi:10.1016/j.ebiom.2026.106283)
Supplement: Full Westem blots [file mmc2.pdf]

Figure 3H

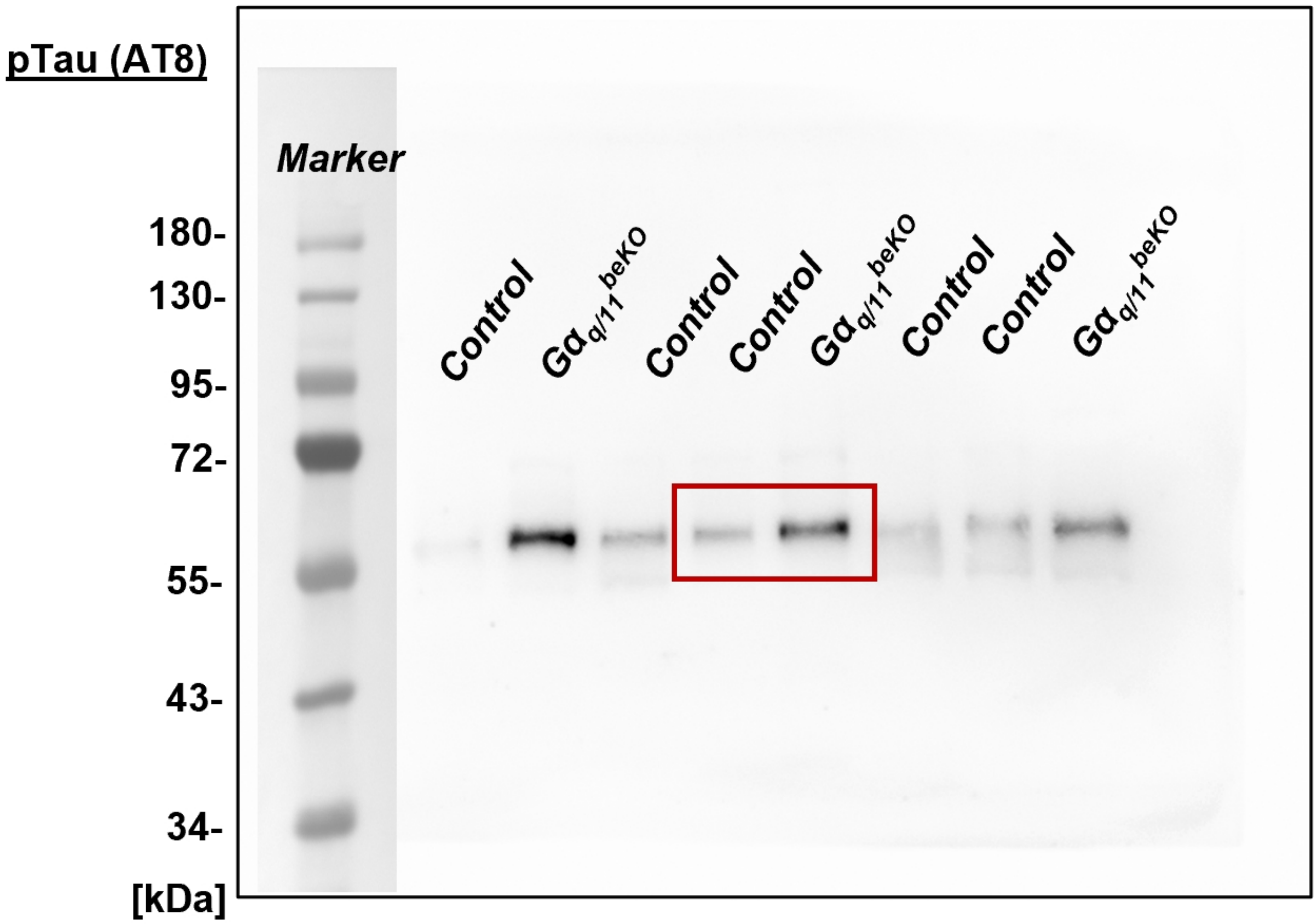

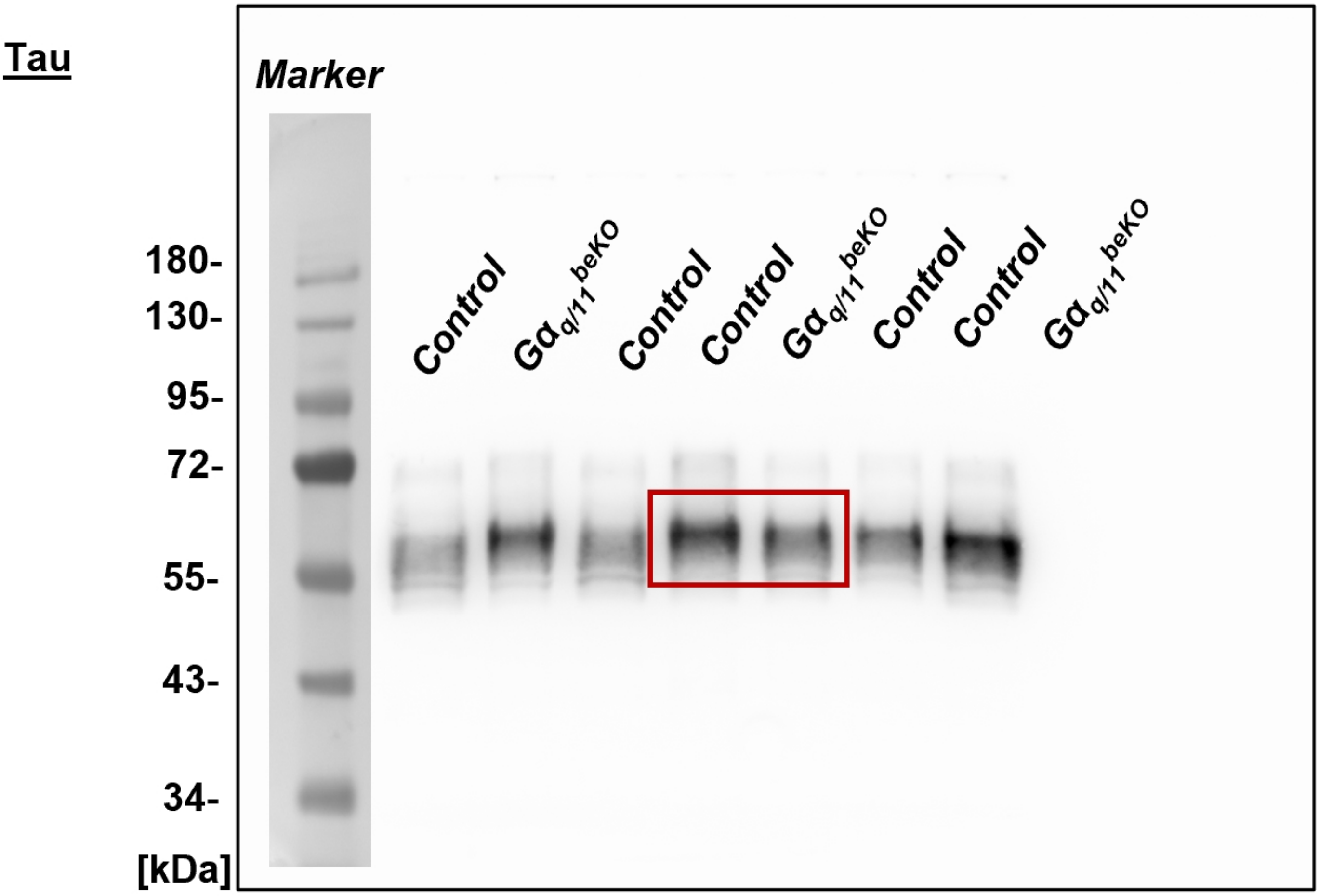

All gels were loaded with 75 µg protein/sample diluted in equal volumes.

Figure 3I

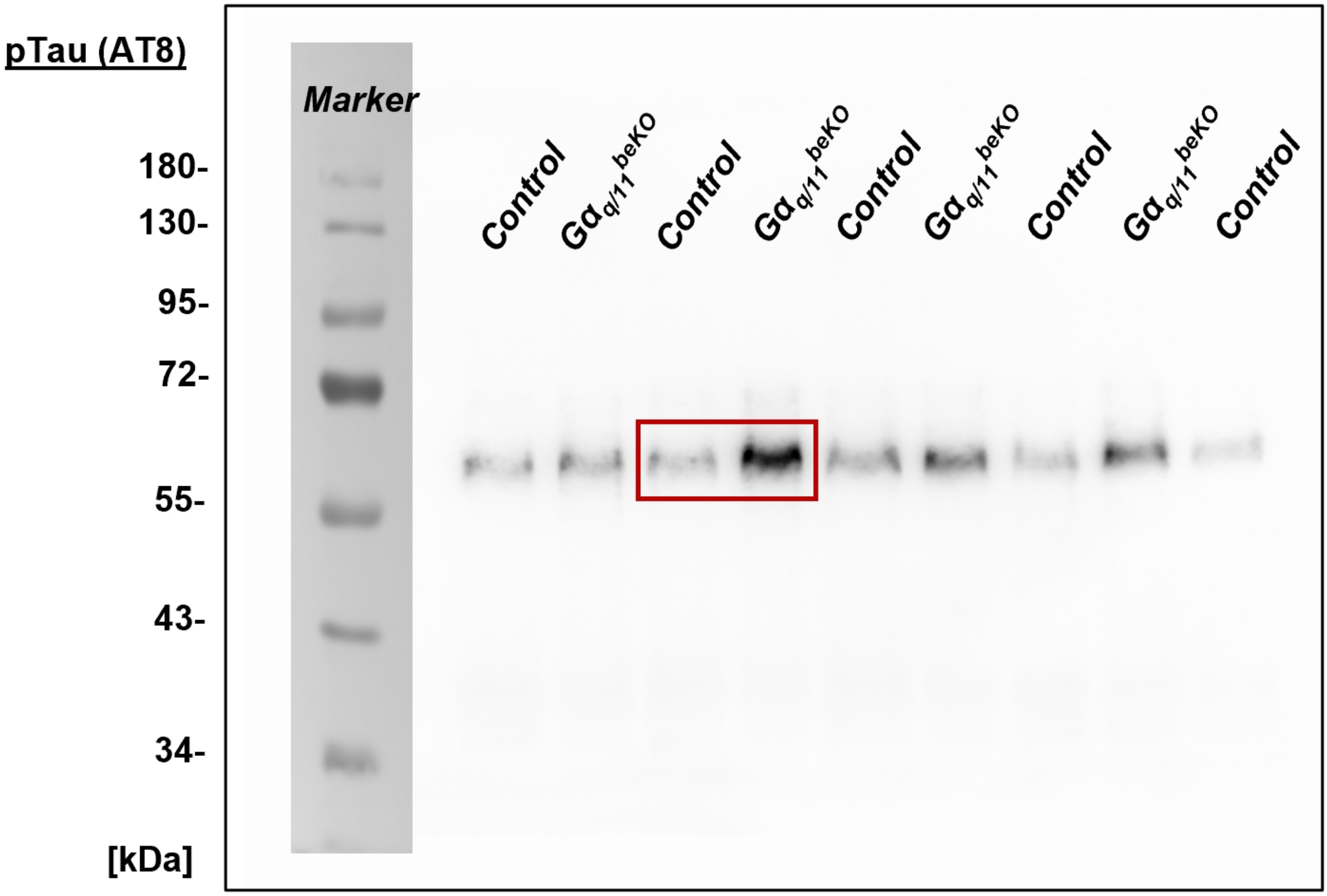

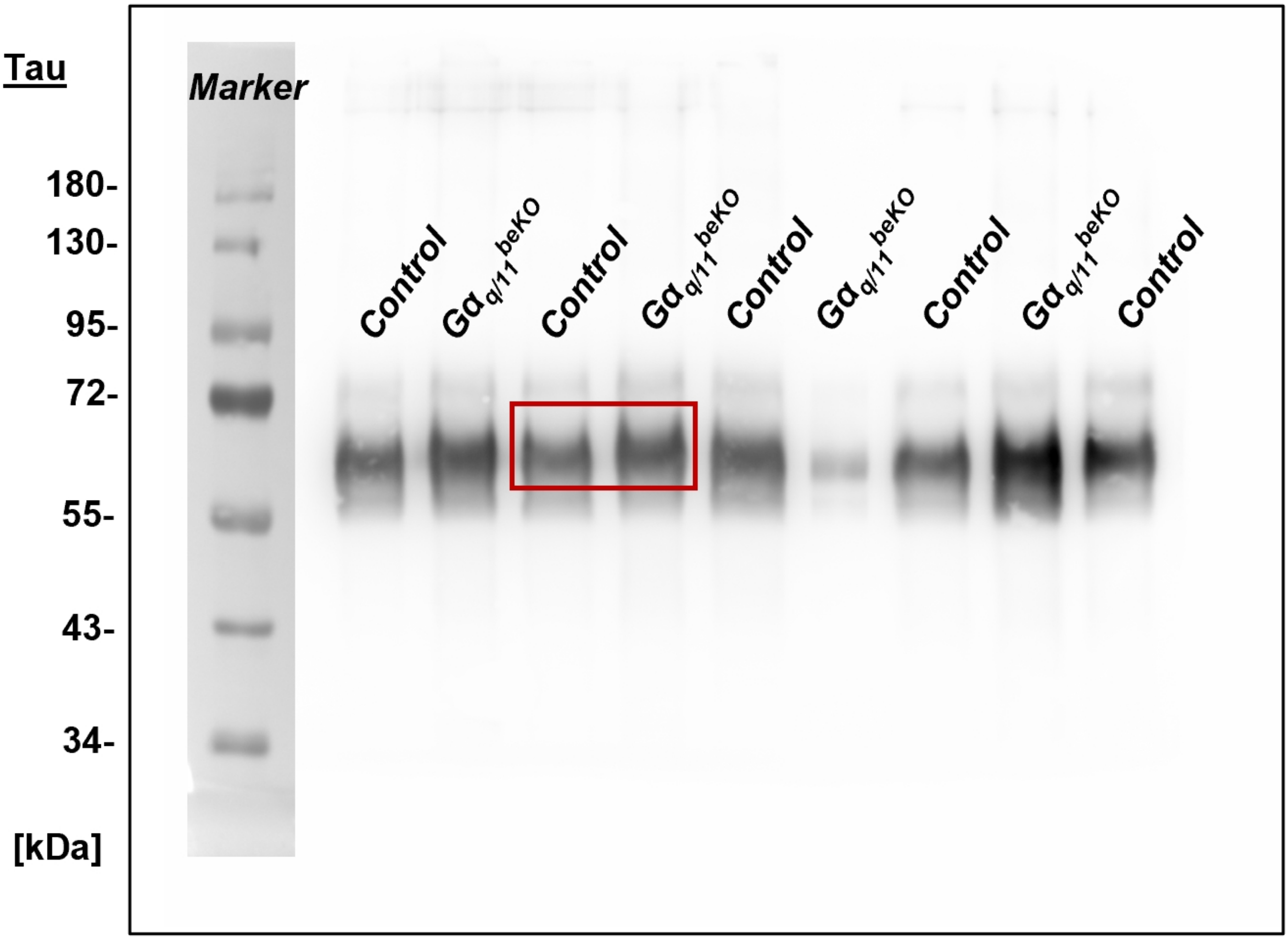

All gels were loaded with 75 µg protein/sample diluted in equal volumes.

Figure 3J

pTau (AT180)

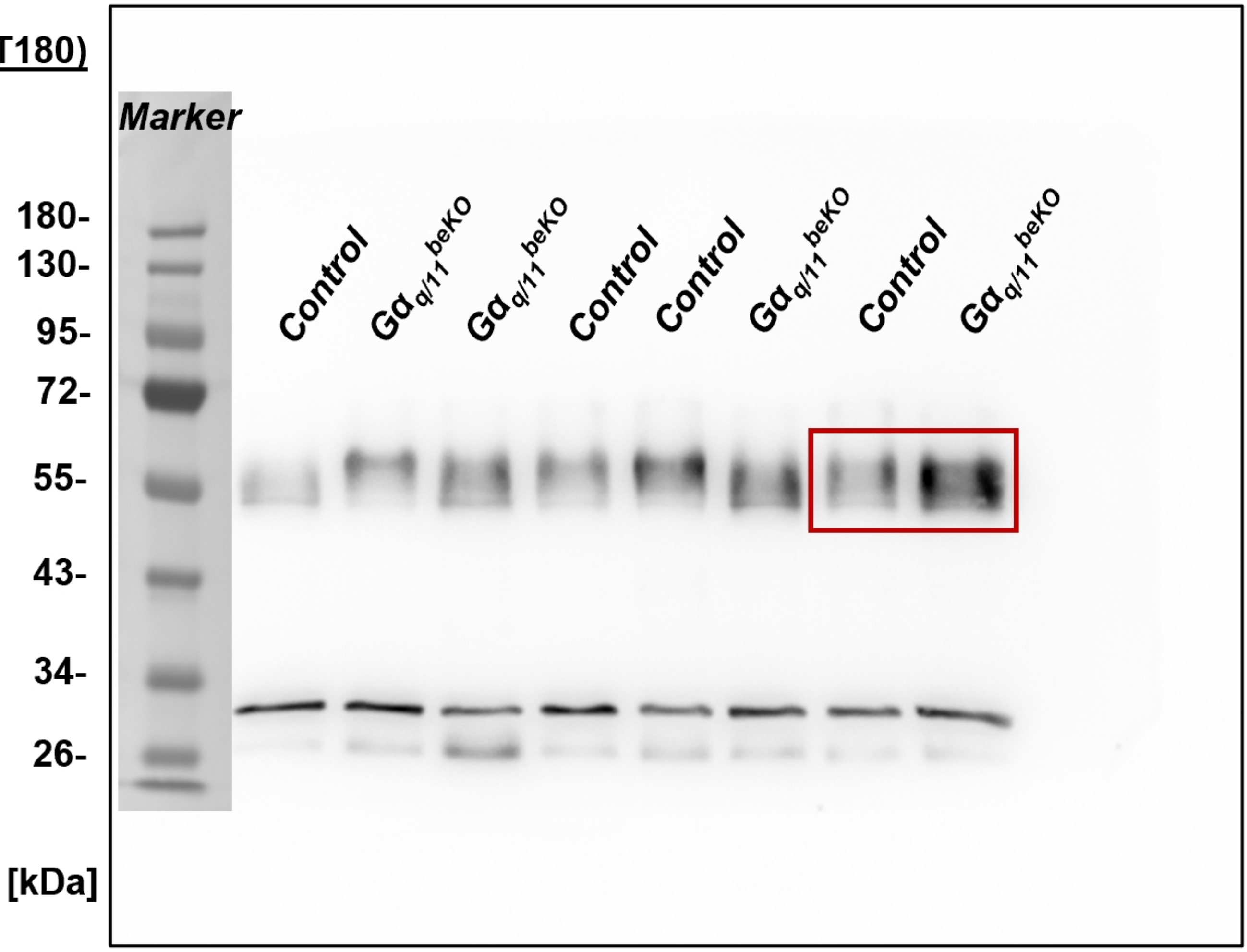

Actin

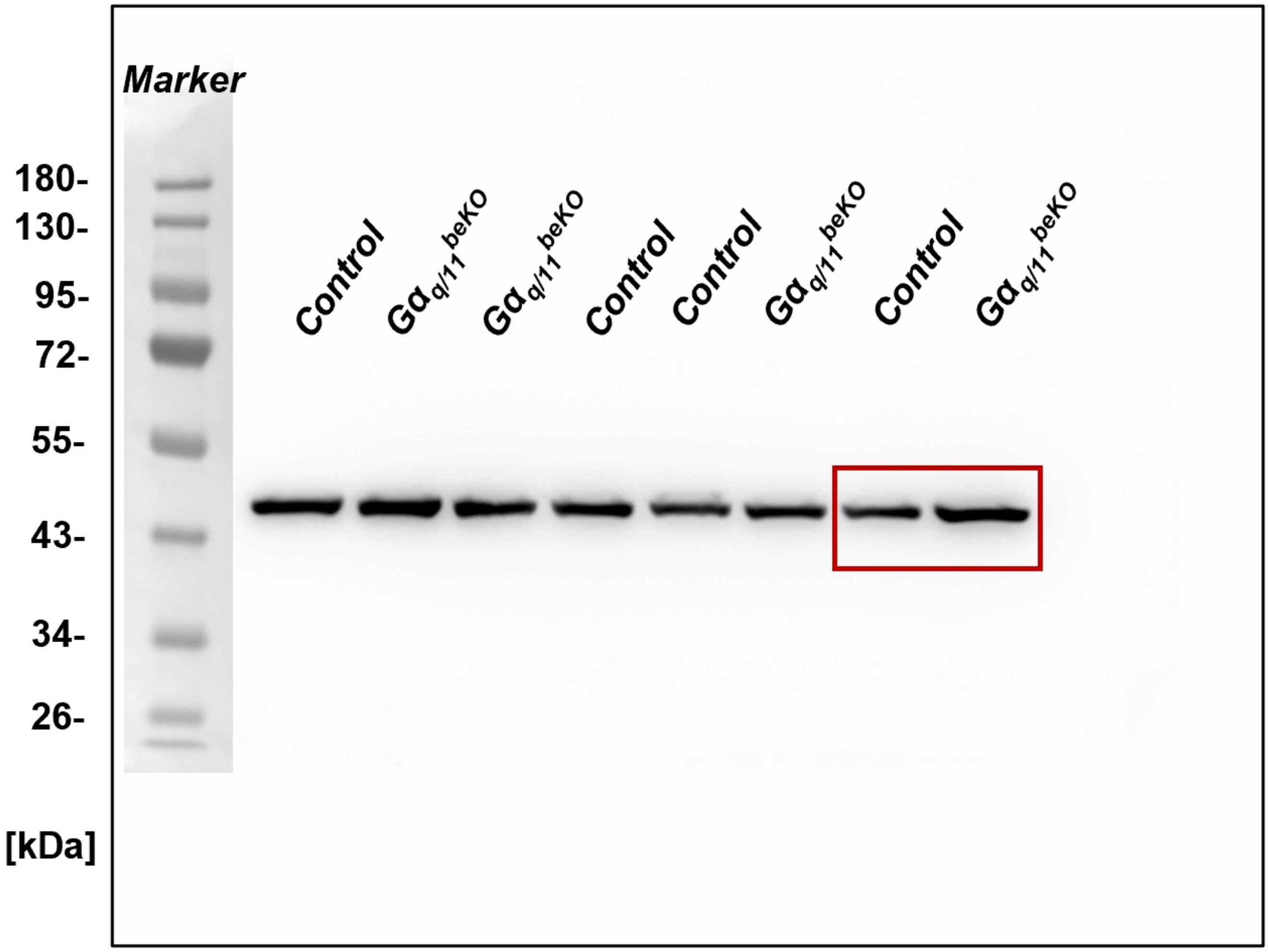

All gels were loaded with 75 µg protein/sample diluted in equal volumes.

Figure 3K

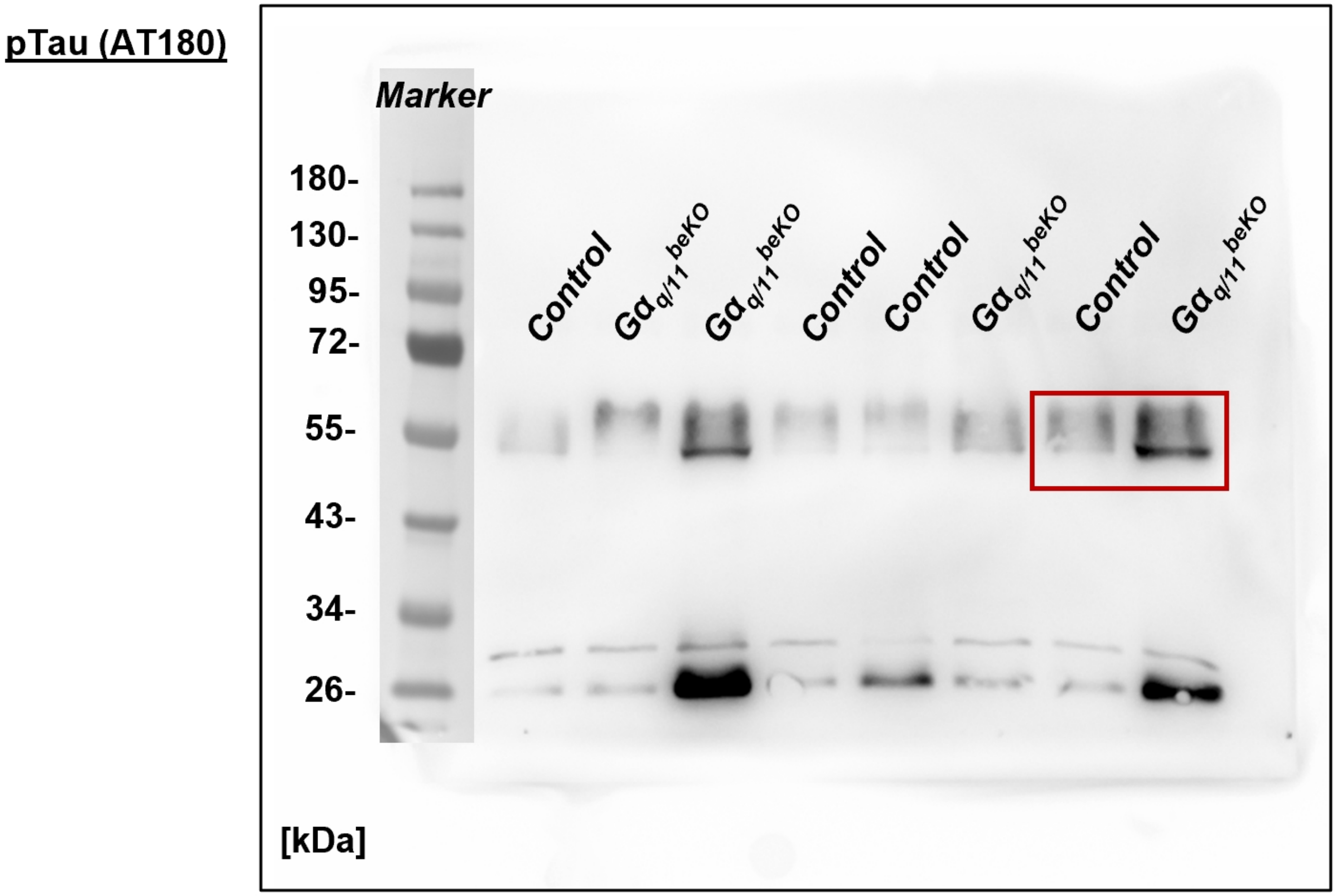

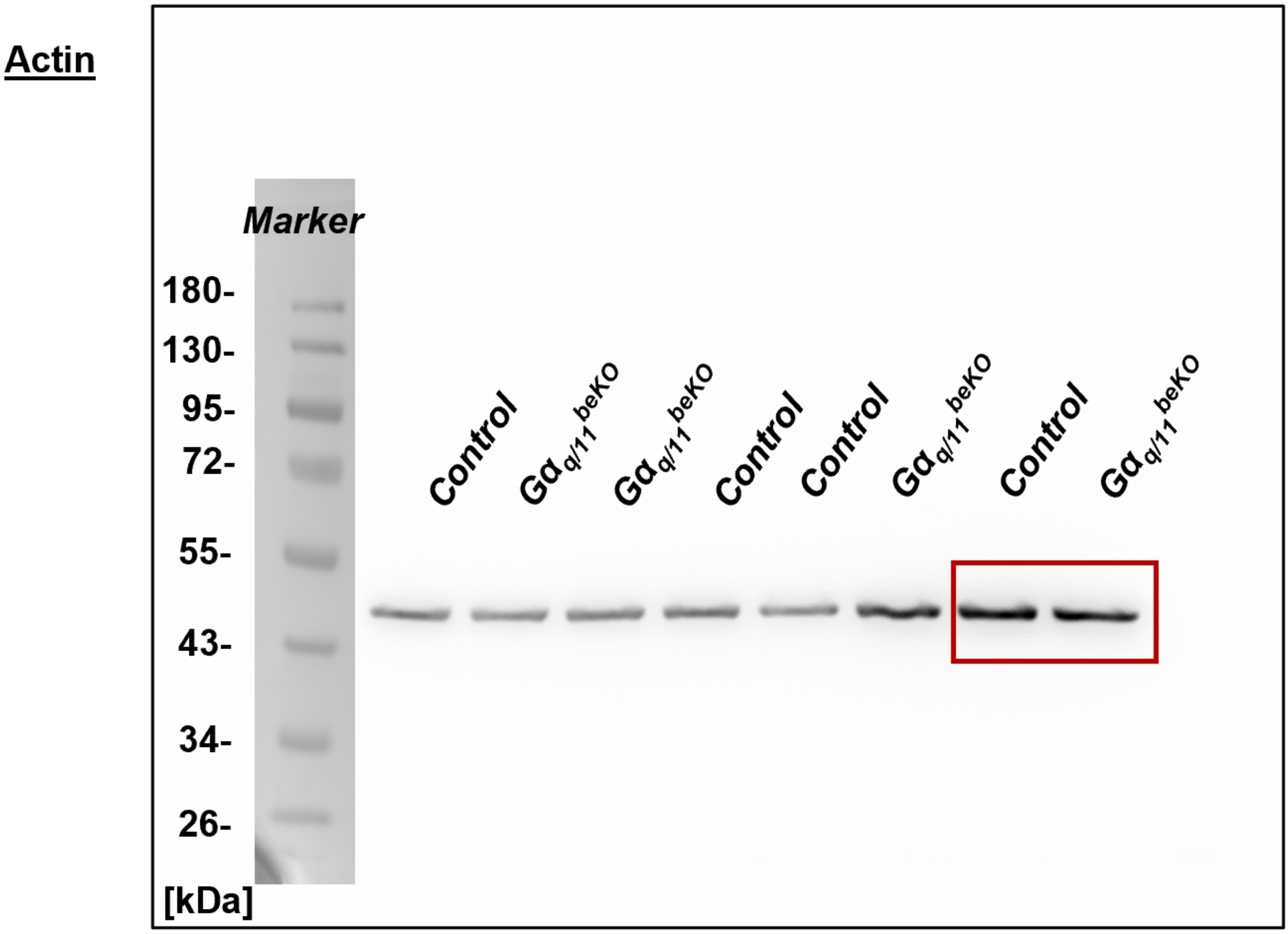

All gels were loaded with 75 µg protein/sample diluted in equal volumes.

Figure 6J - Cortex

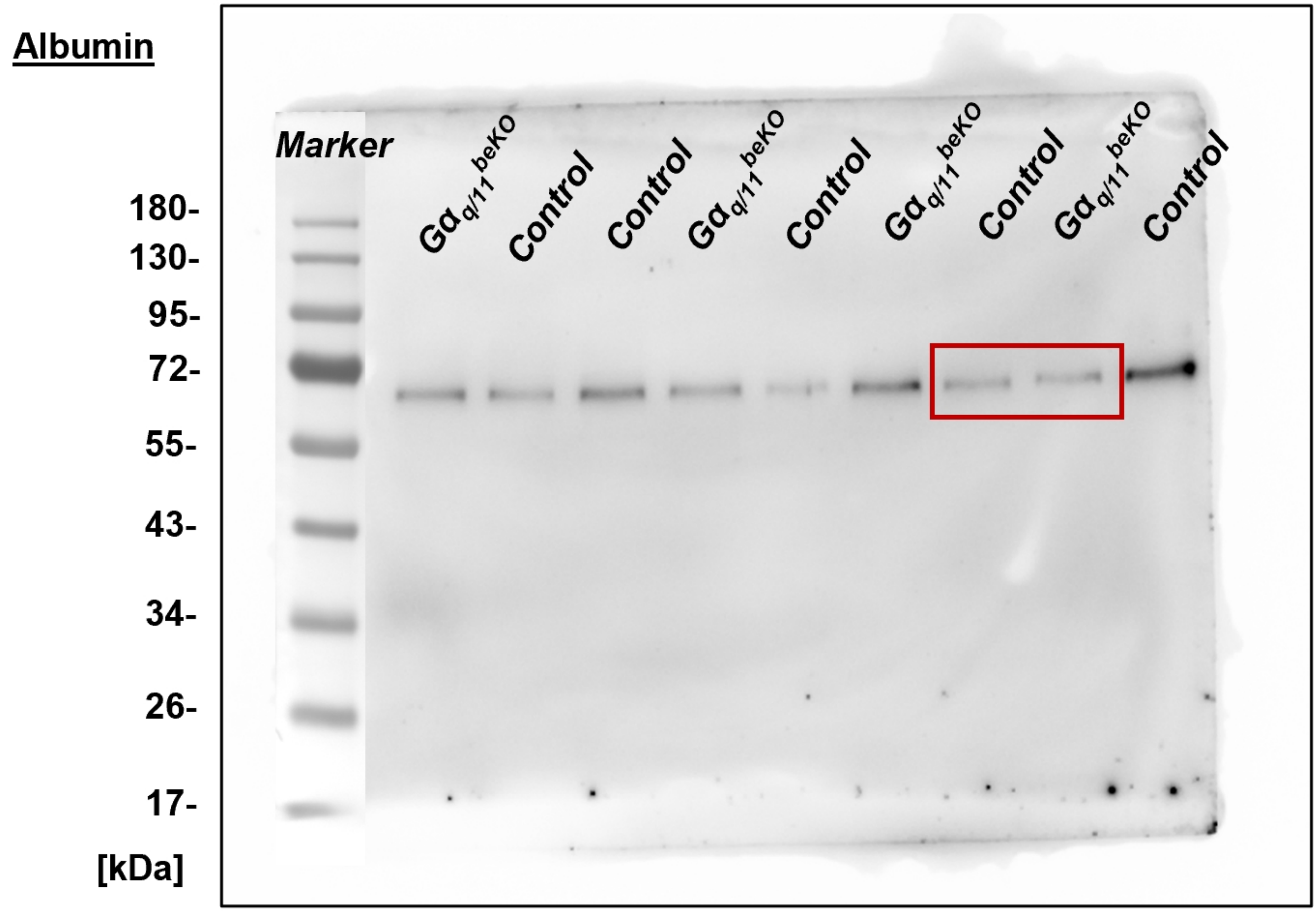

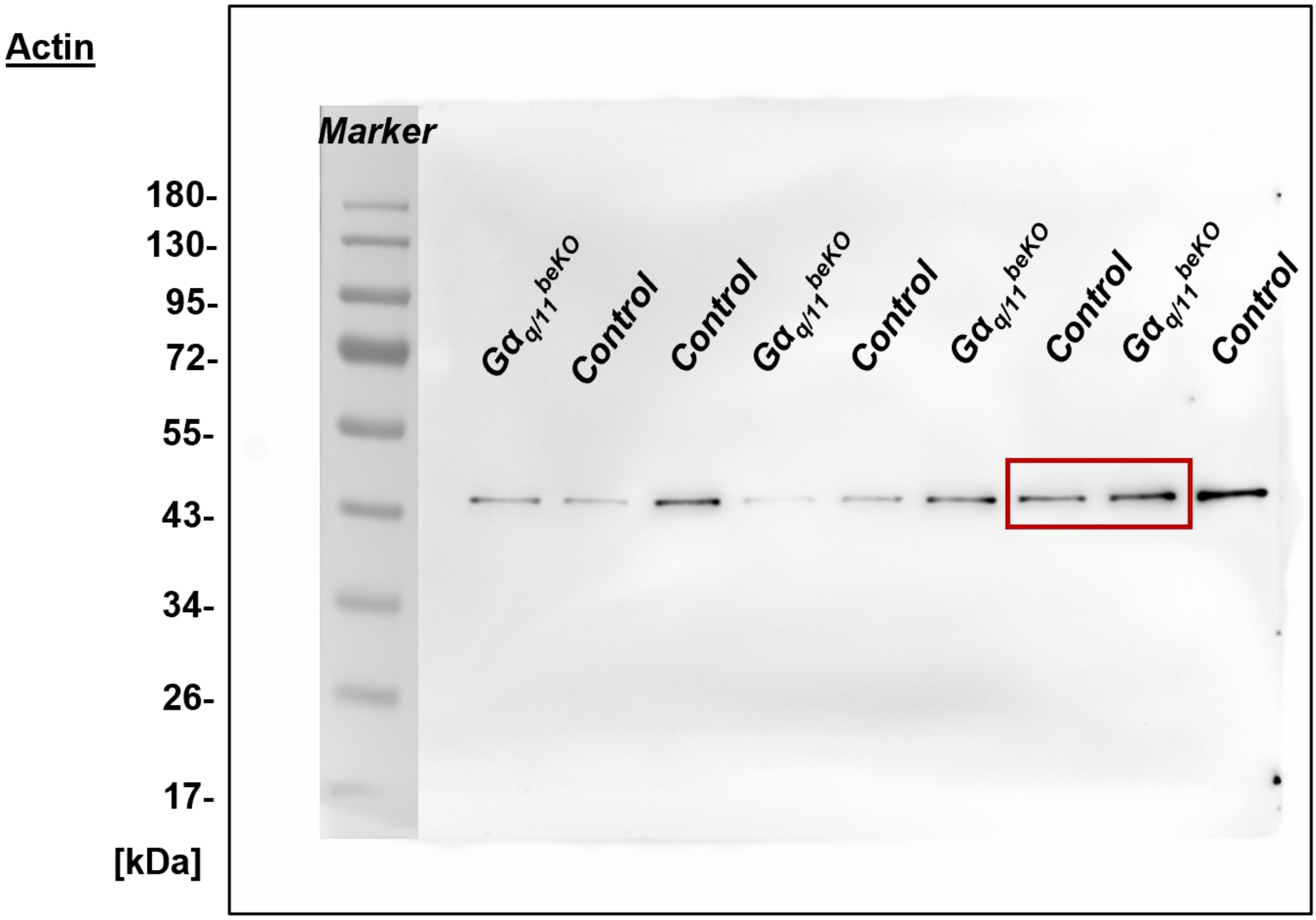

All gels were loaded with 50 µg protein/sample diluted in equal volumes.

Figure 6J - Hippocampus

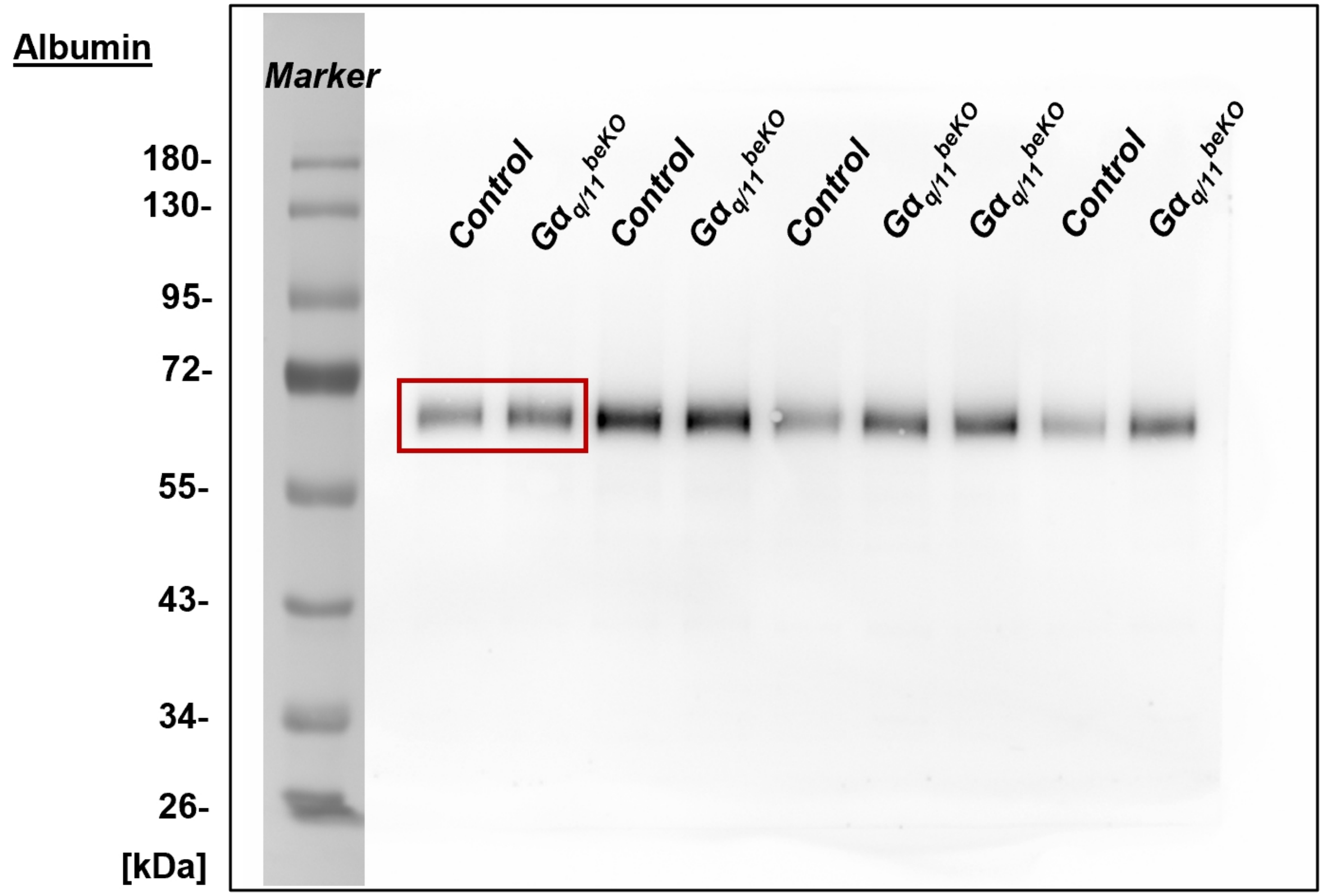

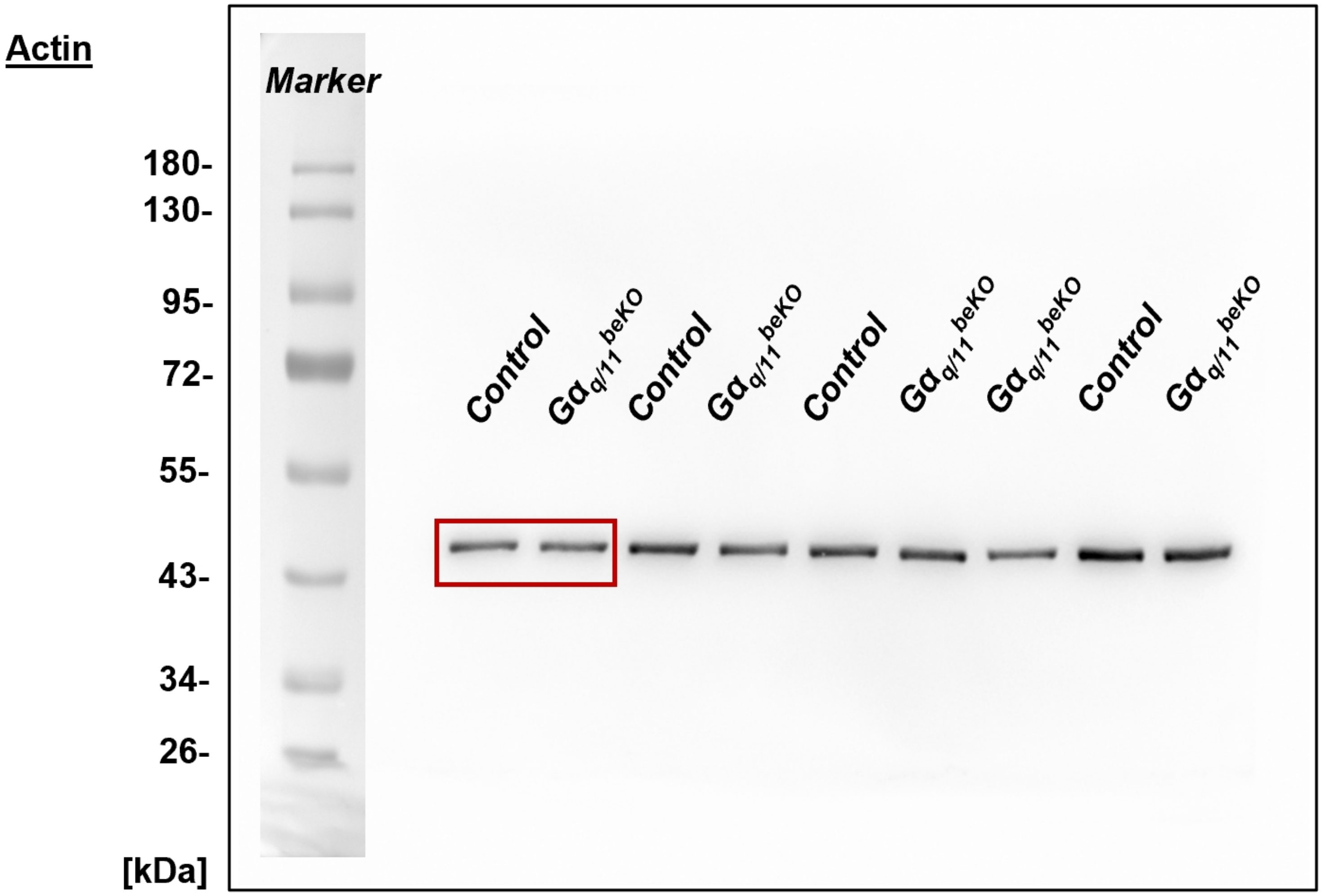

All gels were loaded with 50 µg protein/sample diluted in equal volumes.

Figure 7A

VEGFR2

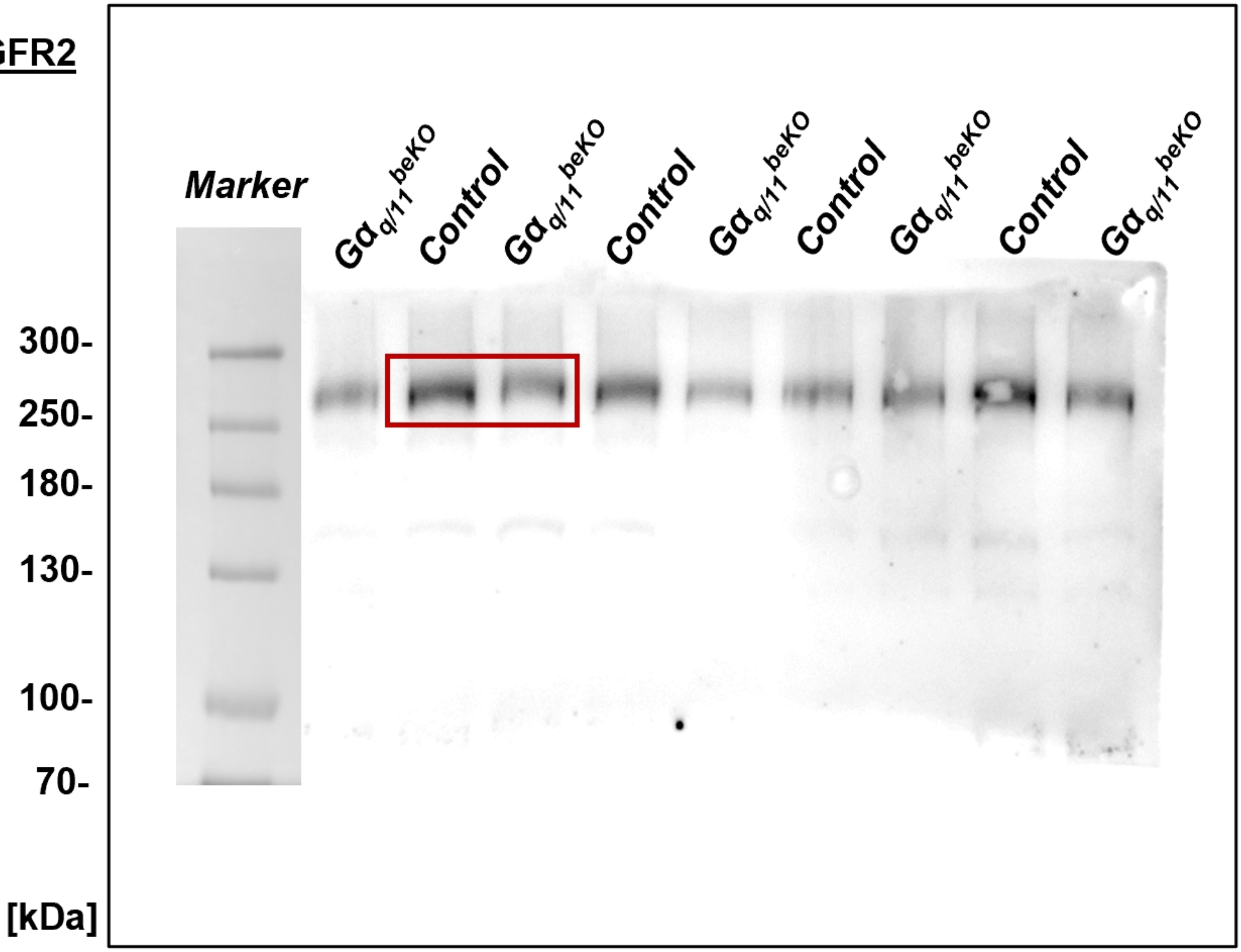

Tubulin

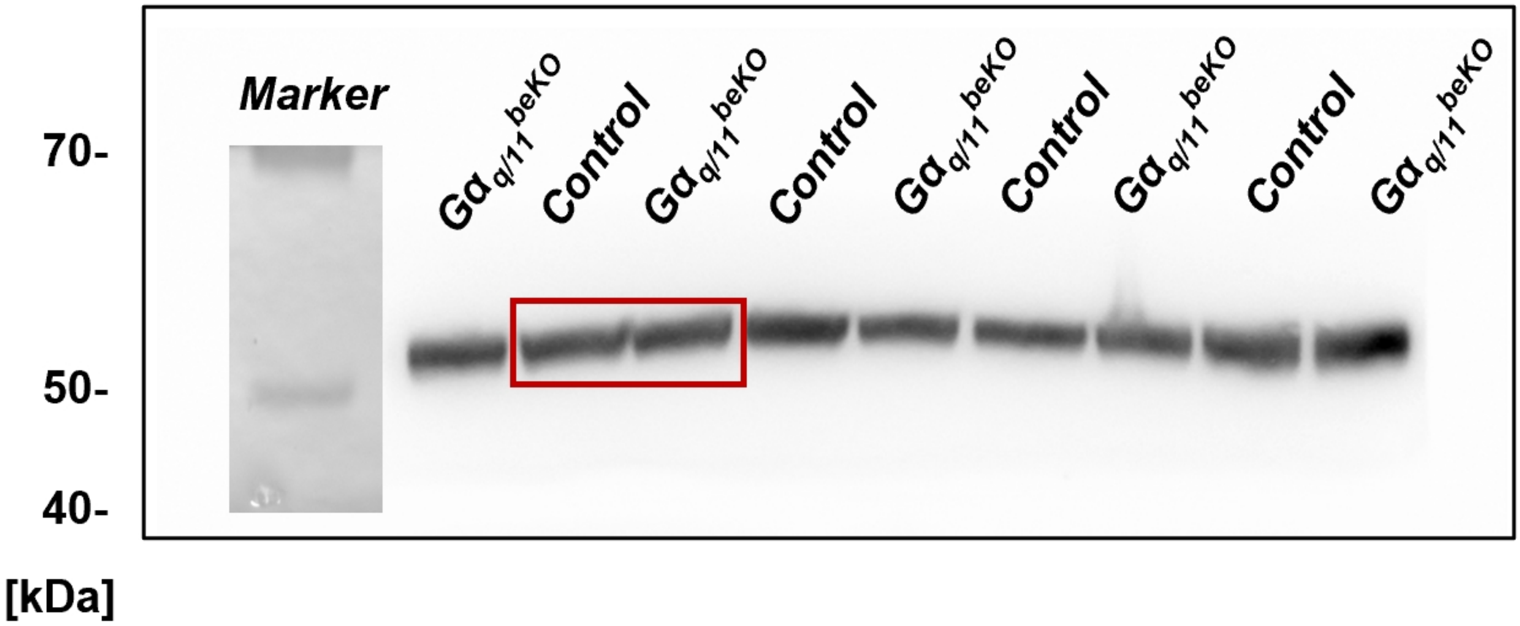

All gels were loaded with 75 µg protein/sample diluted in equal volumes.

Figure 7E

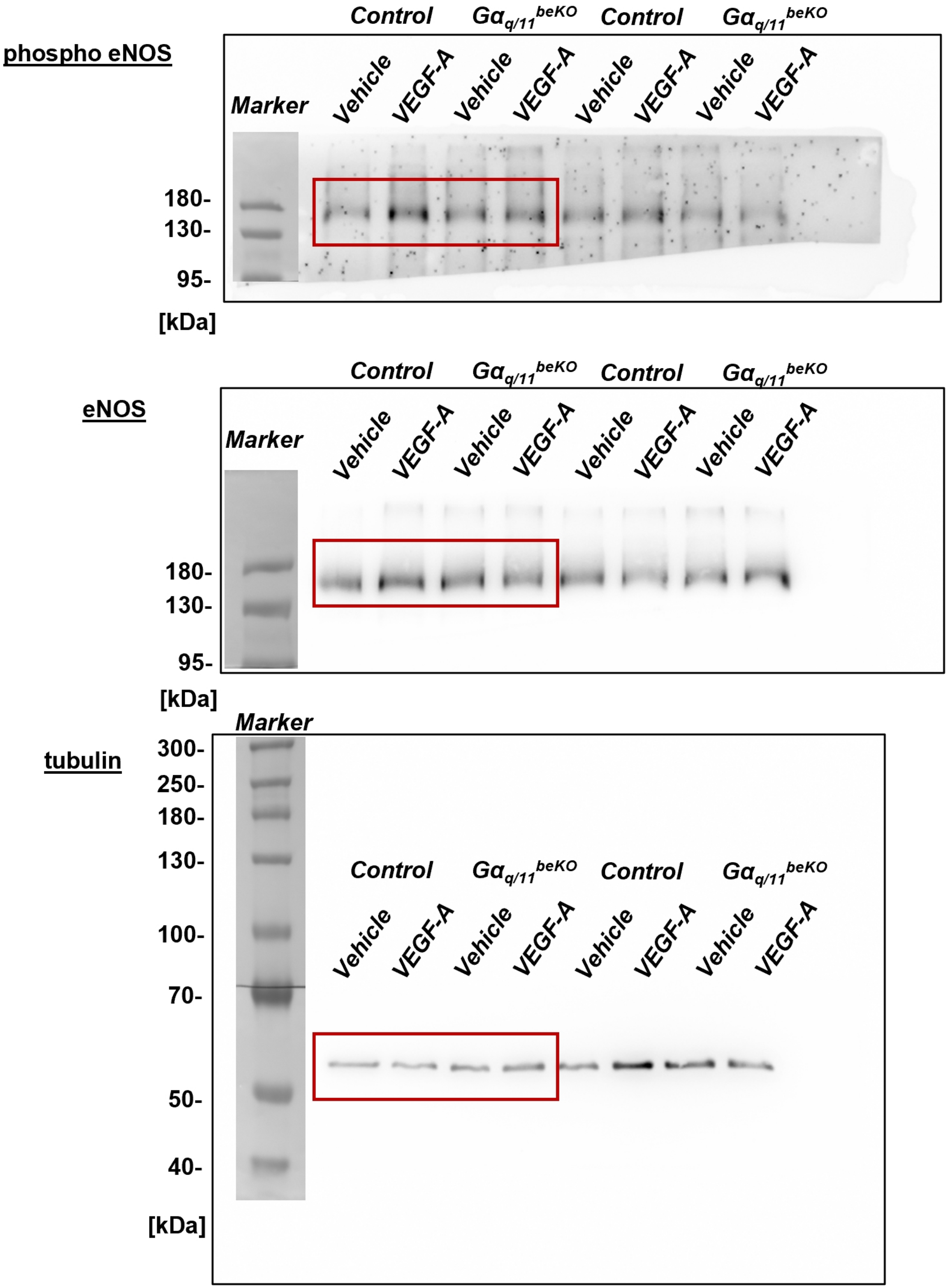

All gels were loaded with 20 µl per sample of the whole lysate of one well of 24-well plate lysed with 100 µl lysis buffer.

Figure 7E

phospho ERK1/2

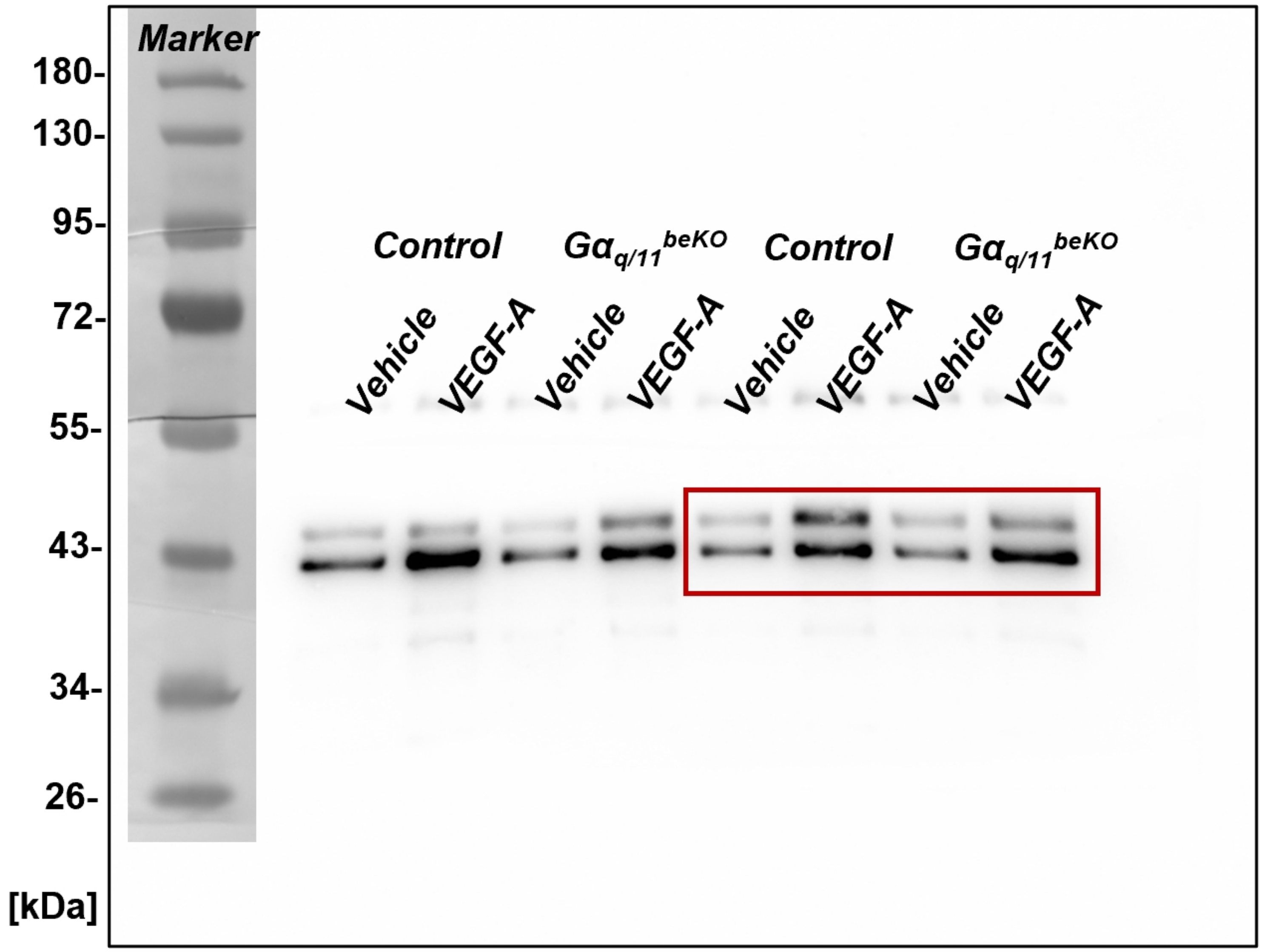

ERK1/2

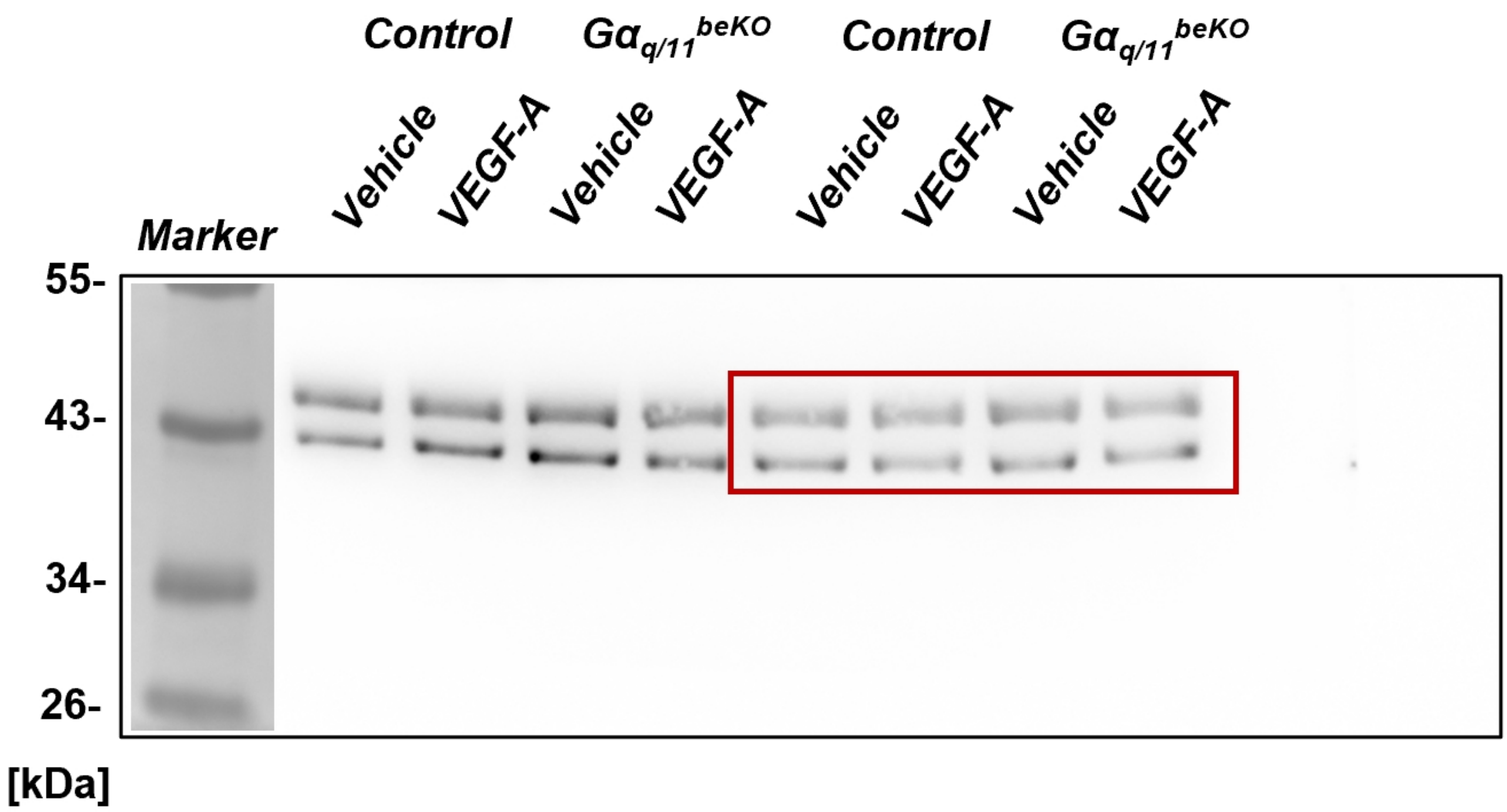

All gels were loaded with 20 µl per sample of the whole lysate of one well of 24-well plate lysed with 100 µl lysis buffer.

Figure 7E

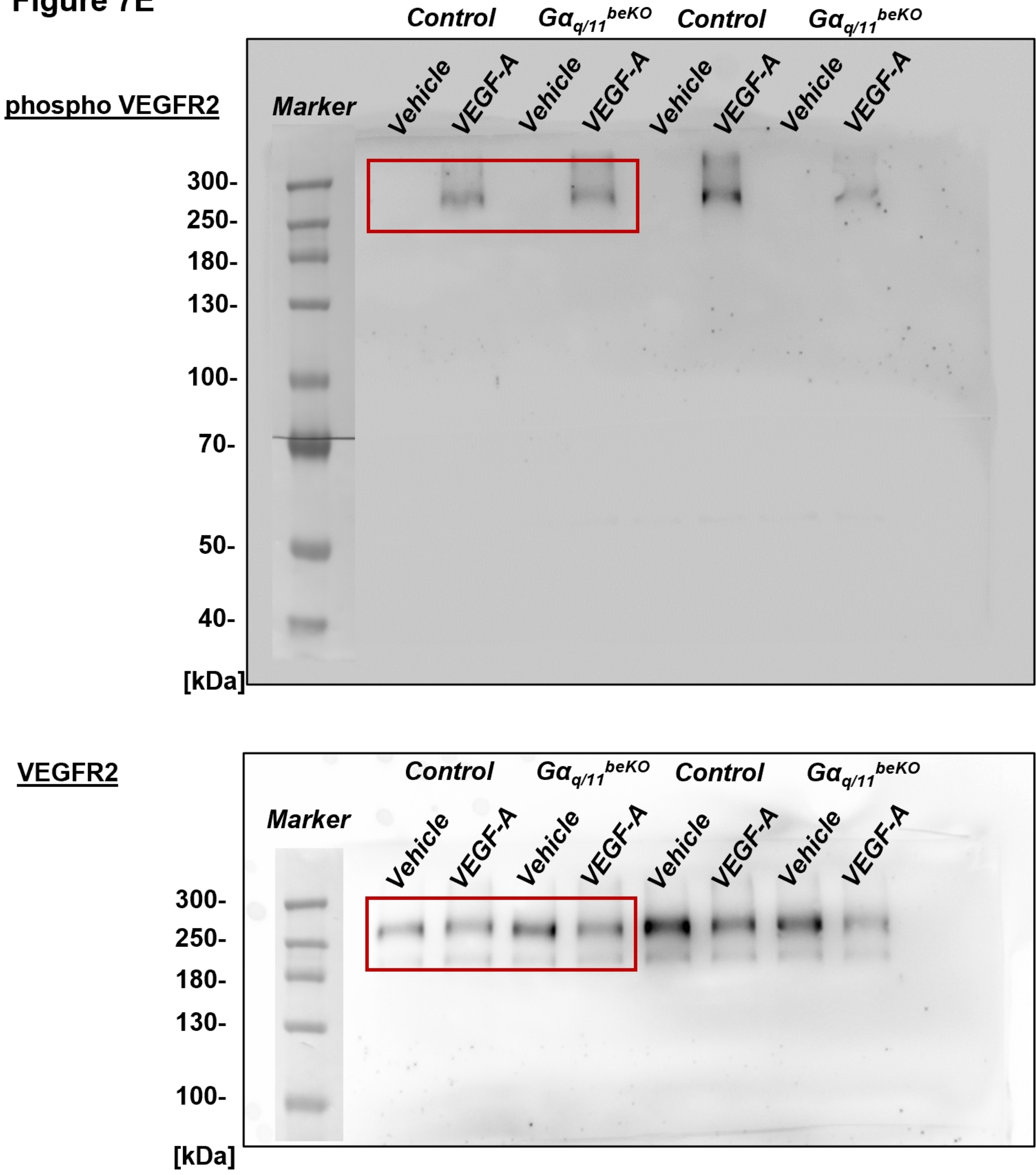

All gels were loaded with 20 µl per sample of the whole lysate of one well of 24-well plate lysed with 100 µl lysis buffer.

Supplemental Figure 8C

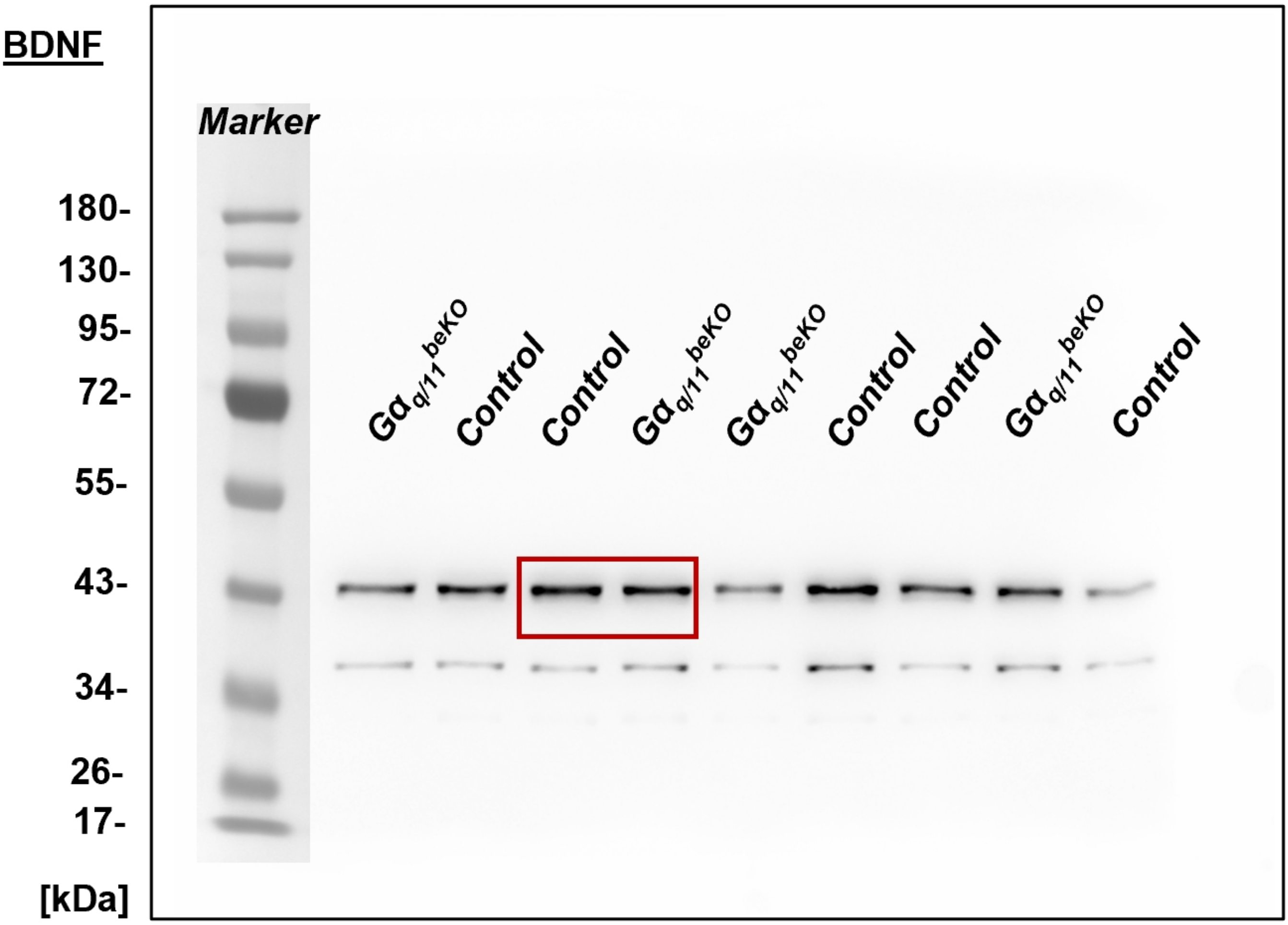

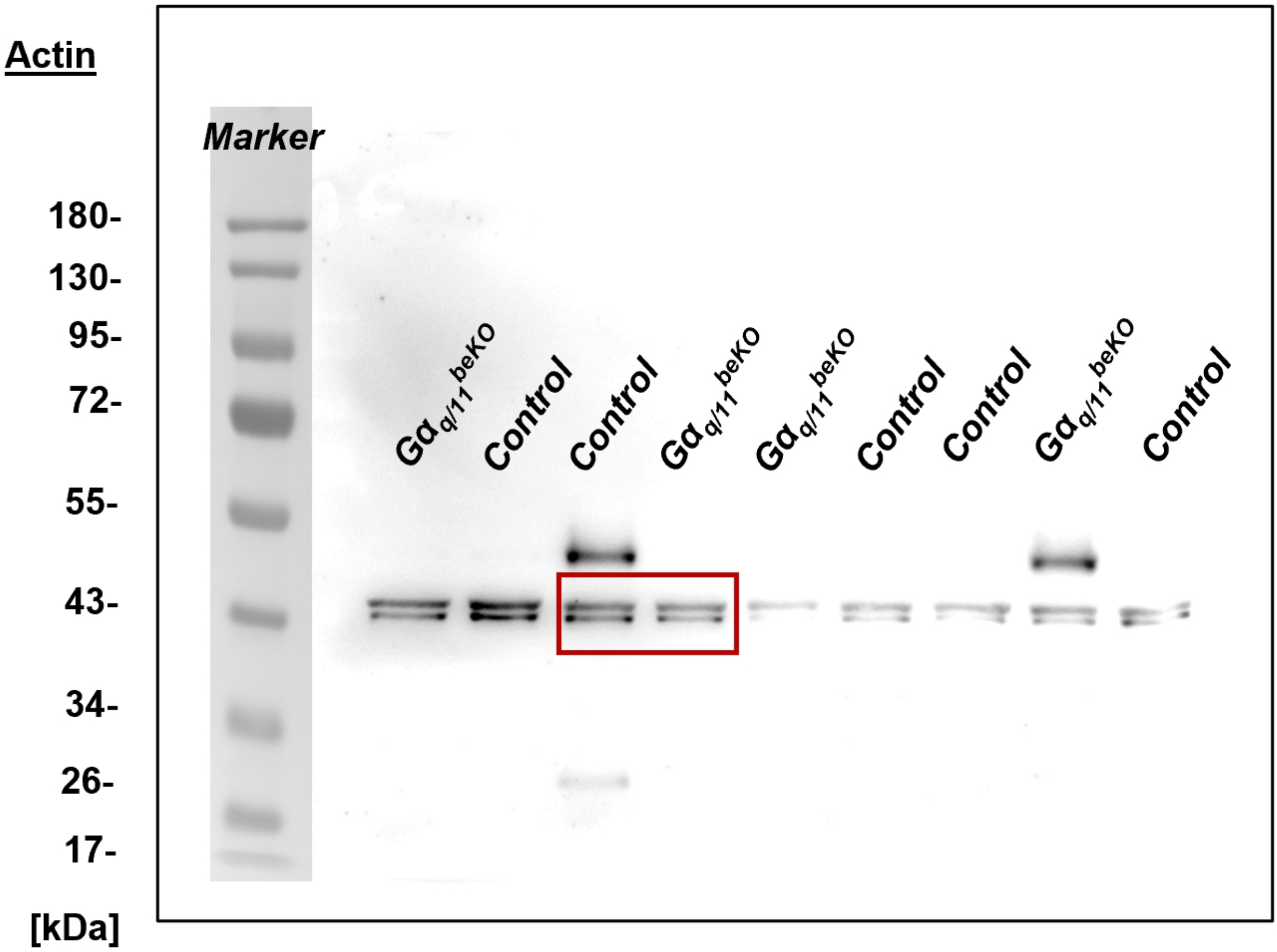

All gels were loaded with 75 µg protein/sample diluted in equal volumes.

Supplemental Figure 8D

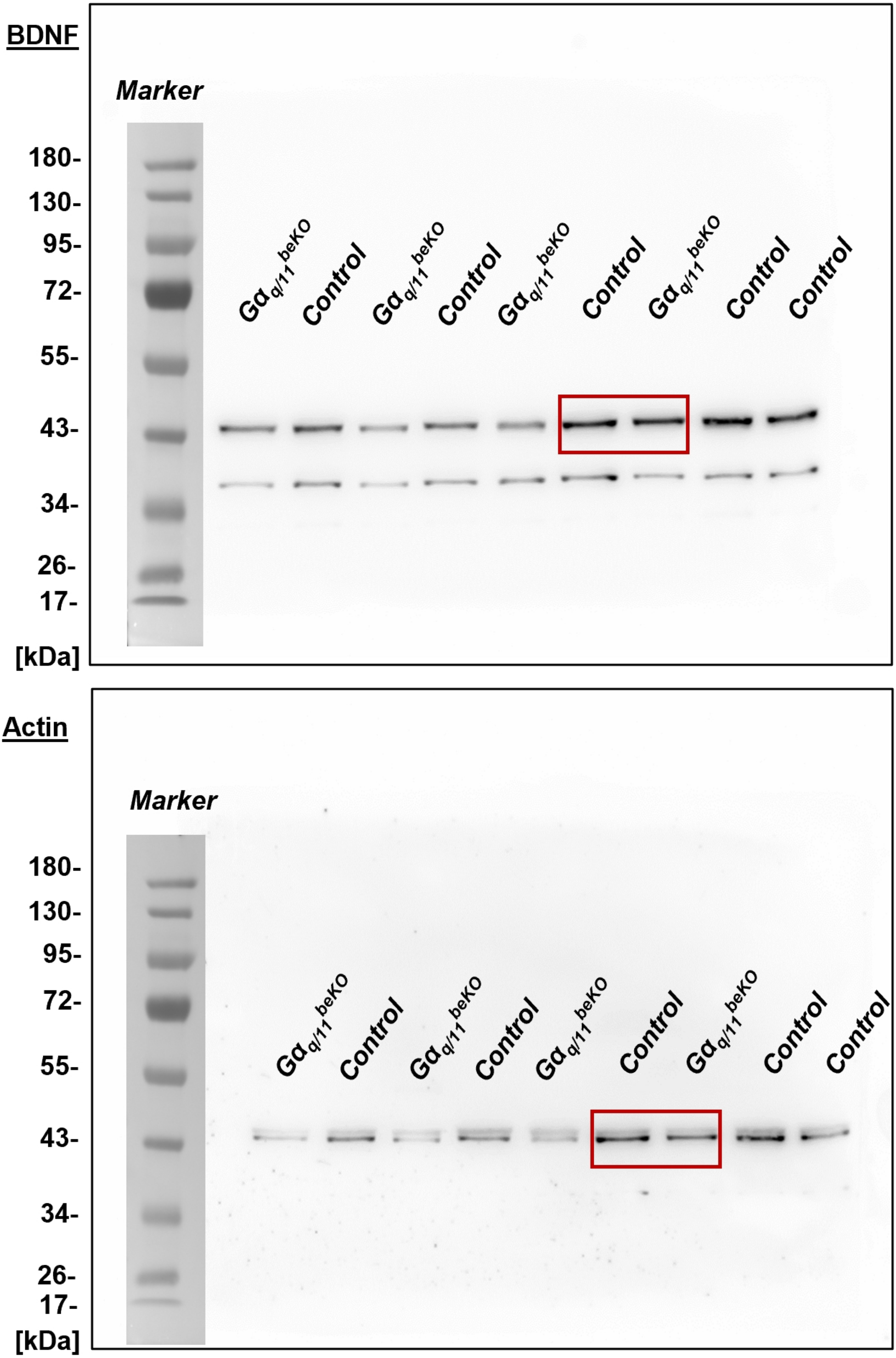

All gels were loaded with 75 µg protein/sample diluted in equal volumes.
